# Hydrogen-Rich Water to Enhance Exercise Performance: A Review of Effects and Mechanisms

**DOI:** 10.3390/metabo14100537

**Published:** 2024-10-07

**Authors:** Qiaorui Zhou, Huixin Li, Ye Zhang, Yirui Zhao, Can Wang, Chang Liu

**Affiliations:** 1 College of Food Science & Nutritional Engineering, China Agricultural University, Beijing 100083, China; 2021306120130@cau.edu.cn; 2 School of Sport Science, Beijing Sport University, Beijing 100084, China; 2021011319@bsu.edu.cn; 3 Sport Coaching College, Beijing Sport University, Beijing 100084, China; 2021010103@bsu.edu.cn; 4 China Ice and Snow Sports College, Beijing Sport University, Beijing 100084, China; 2021010055@bsu.edu.cn

**Keywords:** hydrogen-rich water, athletic performance, oxidative stress, redox homeostasis, exercise physiology

## Abstract

**Background**: Hydrogen-rich water (HRW) has garnered significant interest within the sports and exercise science community due to its selective antioxidant properties. Despite its potential benefits, comprehensive reviews specifically addressing its effects on athletic performance are limited. This review aims to assess the impact of HRW on sports performance and explore the underlying molecular biological mechanisms, with the goal of elucidating how HRW might enhance athletic performance. **Methods**: This review synthesizes research on HRW by examining articles published between 1980 and April 2024 in databases such as PubMed, the Cochrane Library, Embase, Scopus, and Web of Science. **Results**: It highlights HRW’s effects on various aspects of athletic performance, including endurance, strength, sprint times, lunge movements, countermovement jump height, and time to exhaustion. While the precise mechanisms by which HRW affects athletic performance remain unclear, this review investigates its general molecular biological mechanisms beyond the specific context of sports. This provides a theoretical foundation for future research aimed at understanding how HRW can enhance athletic performance. HRW targets the harmful reactive oxygen and nitrogen species produced during intense exercise, thereby reducing oxidative stress—a critical factor in muscle fatigue, inflammation, and diminished athletic performance. HRW helps to scavenge hydroxyl radicals and peroxynitrite, regulate antioxidant enzymes, mitigate lipid peroxidation, reduce inflammation, protect against mitochondrial dysfunction, and modulate cellular signaling pathways. **Conclusions**: In summary, while a few studies have indicated that HRW may not produce significant beneficial effects, the majority of research supports the conclusion that HRW may enhance athletic performance across various sports. The potential mechanisms underlying these benefits are thought to involve HRW’s role as a selective antioxidant, its impact on oxidative stress, and its regulation of redox homeostasis. However, the specific molecular biological mechanisms through which HRW improves athletic performance remain to be fully elucidated.

## 1. Introduction

In recent years, hydrogen-rich water (HRW) has gained recognition as a potential health-promoting beverage, attracting significant attention from athletes, fitness enthusiasts, and researchers alike [1]. The active component of HRW is hydrogen (H_2_), which is believed to offer antioxidant, anti-inflammatory, antiapoptotic, cytoprotective, ergogenic, and recovery-enhancing effects. These properties make HRW a promising adjunctive therapy for optimizing athletic performance and enhancing post-exercise recovery [2].

Despite its growing popularity and anecdotal evidence supporting its efficacy claims, the scientific community is actively working to elucidate the precise mechanisms through which HRW affects exercise physiology. Hydrogen has long been recognized for its nutritional potential across various medical applications, including benefits in oxidative stress-related and inflammatory diseases [3]. However, its application in the realm of sports and physical performance remains relatively unexplored [4]. Given the strict regulations by sports governing bodies like the World Anti-Doping Agency (WADA), which limit the use of many performance-enhancing substances, HRW presents a promising and safe alternative [2]. Its natural occurrence and non-toxic nature make it an attractive option for athletes seeking safe and legal ways to optimize performance and recovery [5].

The suggested benefits of HRW stem mainly from its capacity to deliver molecular hydrogen to the body, serving as a potent antioxidant and signaling molecule [6]. Unlike conventional antioxidants that non-selectively scavenge reactive oxygen species (ROS) and reactive nitrogen species (RNS), hydrogen selectively targets highly reactive and deleterious species such as hydroxyl radicals (^•^OH) and peroxynitrite (ONOO^−^), thereby preserving ROS involved in physiological signaling, which is crucial for cellular homeostasis and adaptation [7]. Recent studies have shown that modulating ROS and subsequently regulating the gas transmitters nitric oxide and carbon monoxide to influence NO-CO metabolism may have beneficial effects on various diseases [8].

Regulation of gene expression: ROS play a role in the regulation of gene expression, particularly in genes involved in stress responses and antioxidant production [9]. By maintaining physiological levels of ROS, hydrogen can aid in the proper regulation of these genes, supporting cellular defense mechanisms and resilience against oxidative stress [10]. Overall, these beneficial properties not only protect cells from oxidative damage but also support critical signaling pathways that promote health, adaptation, and recovery.

The potential effects of HRW on exercise physiology are multifaceted, influencing a wide range of physiological processes encompassing energy metabolism [11], oxidative stress modulation [12], inflammation regulation [13], cell signaling modulation [14], and recovery facilitation mechanisms [15,16]. By regulating these key pathways, HRW offers an alternative approach to optimizing athletic performance [17], mitigating exercise-induced muscle damage [2] and accelerating post-exercise recovery [18].

Despite growing interest in this area, several fundamental questions remain unanswered [1]. A primary concern is determining which specific exercise patterns—such as sprint intervals, 30-s rowing distances, or 30-m sprint times—derive the most benefit from HRW supplementation [19]. It is well established that sports encompass a diverse range of activities, each requiring distinct skills such as cardiorespiratory fitness, muscular strength, muscular endurance, flexibility, agility, coordination, power, reaction time, and speed [20]. However, the extent to which HRW can enhance these specific skills remains unclear and warrants further investigation. Additionally, to effectively develop HRW as a sports supplement and promote its use among a broader athletic audience, it is crucial to identify the specific physiological advantages that might enhance athletic performance [21].

Meanwhile, it is essential to investigate the molecular biological mechanisms underlying the effects of HRW. Understanding these mechanisms will enable future sports researchers to design targeted interventions and optimize athletic performance more effectively. This integrated approach could ultimately lead to the development of more effective strategies for enhancing performance across various sports disciplines.

This review aims to evaluate the current state of knowledge on the application, effects, and mechanisms of HRW in sports. By reviewing the latest findings from double-blind studies in real-world athletic populations and mechanistic and clinical studies, including those in molecular biology, we seek to provide a nuanced understanding of the potential role of HRW in exercise. By investigating its physiological effects, elucidating its molecular mechanisms of action, and discussing its practical implications for athletes and active individuals, this review aims to provide valuable insights and inspiration for future research in the dynamic field of HRW and sports science.

**Figure 1:**
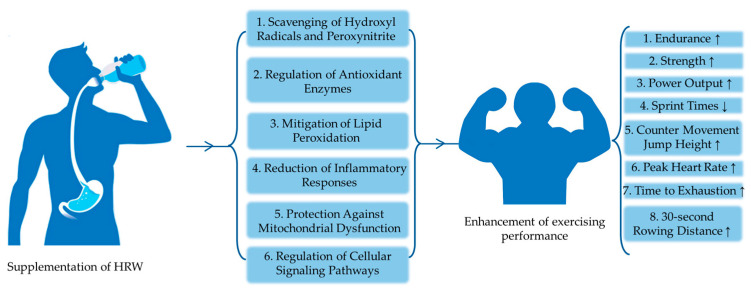
Potential effects of HRW and its role in promoting muscle health and enhancing athletic performance.

In general, the main goal of this paper is to explore the effects of HRW during exercise, elucidate its potential mechanisms of action, discuss its potential applications and challenges, and outline future research directions in this rapidly evolving field. By clarifying the multifaceted role of HRW in exercise physiology, we aim to promote evidence-based practice and innovation in the fields of sports science and sports medicine (Figure 1).

## 2. Methods

This narrative review employed a literature search spanning articles published between 1980 and April 2024, sourced from databases including PubMed, the Cochrane Library, Embase, Scopus, and Web of Science. The search aimed to encompass all the studies examining the impact of HRW on sport performance during this period. Search terms included “HRW”, “hydrogen-rich water”, “hydrogen water”, and “athletic performance”, “sport performance”, “exercise performance”, combined using the Boolean operators “AND” and “OR”. The database search was performed by authors QZ and CW. Subsequently, the retrieved articles were exported to an Excel^®^ sheet, where duplicate entries were identified and removed. Any discrepancies were resolved through a discussion between the lead reviewer and a third reviewer (CL) [22].

## 3. The Effects of HRW on Exercise Performance Enhancement

For a considerable time, scientists have held a prevailing belief in the potential of HRW to enhance exercise recovery [23] and mitigate fatigue [18]. Nonetheless, research directly validating its ability to significantly augment sports performance and enhance competitive capabilities remains limited [1]. Enhancing sports performance and the competitive edge is of paramount importance in the field, surpassing indirect benefits such as improved exercise recovery and fatigue resistance. Therefore, we begin with a meticulous review of the existing but limited literature on this topic. Table 1 presents a summary of the research findings on the effects of HRW in exercise.

### 3.1. Significant Outcomes

Several studies, which are detailed below, have investigated the potential benefits of HRW supplementation on exercise performance, documenting improvements in endurance, strength, sprint times, lunge movements, countermovement jump height, peak power output, repetitive sprints, peak heart rate, time to exhaustion, rate of perceived exertion, 30-s rowing distance, mean power, and 30-m sprint time. These findings suggest that HRW may serve as a valuable ergogenic aid, potentially enhancing multiple dimensions of athletic performance.

A randomized, double-blind, placebo-controlled crossover study was conducted in 16 professional male football players, aged 18.8 ± 1.2 years. The study comprised two indoor tests in which the athletes performed 15 × 30 m track and field sprints, with a 20-s recovery period between sprints, and a 1-week washout period between the two test sessions. The HRW group received an initial dose of 420 mL 120 min before the sprints, followed by a second 420 mL dose at 60 min, and two final doses of 210 mL at 15 and 5 min before the repeated sprints. During the washout period, participants were instructed to avoid consuming caffeine-containing beverages, such as coffee or tea, and any other substances that could potentially affect the physiological, biochemical, and perceptual outcomes, as well as to abstain from alcohol and strenuous physical activity. Sprint times were recorded at the 15 m and 30 m marks. The results indicated that sprint times for the 14th and 15th 15 m sprints were significantly faster following the administration of 1260 mL of HRW compared with the control group, who received 1260 mL of purified water, showing improvements of 3.4% (HRW: 2.57 ± 0.12 s; placebo: 2.66 ± 0.15 s) and 2.7% (HRW: 2.57 ± 0.09 s; placebo: 2.64 ± 0.13 s), respectively, during the time window of 8:30–11:00. Additionally, the HRW group demonstrated a significant 1.9% (HRW: 4.54 ± 0.14 s; placebo: 4.63 ± 0.17 s) enhancement in the 30 m sprint time at the final sprint [18].

In a randomized, double-blind, placebo-controlled crossover study, 12 men with an average age of 23.8 ± 1.9 years performed half squats, knee flexion, and extension exercises with a load set at 70% of their one-repetition maximum (1RM), completing three sets of 10 repetitions each. Additionally, they performed lunges at 30% of their body weight for three sets with 20 repetitions per set. Measurements of time, lactate levels, and perceived exertion were taken midway through and immediately after the exercise regimen. Furthermore, markers such as creatine kinase levels, visual analog scale ratings for muscle soreness, counter motor jump performance, and heart rate variability were evaluated before the training session and at 30 min, 6 h, and 24 hours post recovery. When comparing HRW with placebo (the HRW group consumed 1260 mL of HRW at one time before the experiment, while the placebo group consumed 1260 mL of purified water under the same conditions), the lunge movements exhibited a significantly greater speed (*p* < 0.001) when supplemented with HRW. Additionally, HRW supplementation led to a reduction in lactate levels both during and immediately after exercise (HRW: 5.3 ± 2.1 and 5.1 ± 2.2 mmol·L^−1^, respectively; placebo: 6.5 ± 1.8 and 6.3 ± 2.2 mmol·L^−1^, respectively; *p* ≤ 0.008). The visual analog scale scores for muscle soreness were significantly lower 24 h after recovery with HRW compared with placebo (26 ± 11 vs. 41 ± 20 mm; *p* = 0.002). These findings suggest that acute intermittent hydration with HRW enhances muscle function, mitigates the lactate response, and alleviates delayed-onset muscle soreness [23].

A study was conducted in eight female participants (mean ± SD: age 21.5 ± 5.0 years; maximum oxygen consumption 45.0 ± 2.5 mL·kg^−1^·min^−1^) and four male participants (age 18.9 ± 1.3 years; maximum oxygen consumption 52.2 ± 1.7 mL·kg^−1^·min^−1^). This investigation employed a randomized, double-blind, placebo-controlled, crossover trial. Each participant underwent a 12 × 50 m sprint in the morning followed by a 400 m athletic performance in the afternoon. Three days prior to the experiment, and on the day of the experiment, participants consumed either HRW or placebo (1260 mL/day as a baseline three days prior to the experiment, and 2520 mL as the double dose on the day of the experiment, while the placebo group consumed the same amounts of purified water). Muscle performance (measured by countermovement jump), muscle damage (assessed by creatine kinase levels), and muscle soreness (evaluated using a 100 mm visual analog scale) were evaluated on the day of the experiment, as well as at 12 and 24 h after the afternoon training session. The results indicated that compared with placebo, HRW led to improvements in countermovement jump height (30.7 ± 5.5 cm vs. 29.8 ± 5.8 cm; *p* = 0.014) and reductions in creatine kinase blood activity (156 ± 63 UL^−1^ vs. 190 ± 64 UL^−1^; *p* = 0.043), as well as decreased muscle soreness (34 ± 12 mm vs. 42 ± 12 mm; *p* = 0.045) at the 12-h mark post afternoon training [2].

In a separate investigation, eight well-trained male cyclists (mean ± SD: age: 41 ± 7 years; weight: 72.3 ± 4.4 kg; height: 1.77 ± 0.04 m; maximal oxygen uptake (ṼO_2_max): 52.6 ± 4.4 mL·kg^−1^·min^−1^) were enlisted in a randomized, double-blind, placebo-controlled crossover study. Participants consumed either 2 L per day of placebo plain water (pH 7.6; oxidation/reduction potential (ORP): +230 mV; free hydrogen content: 0 ppb) or HRW (pH 9.8; ORP: −180 mV; free hydrogen content: 450 ppb. Assessments were conducted at baseline and following each two-week treatment phase, with the treatment allocation counterbalanced and order randomized. The 30-min intermittent cycling trial comprised 10 blocks, each consisting of 3 min at 40% ṼO_2_max, 60 s at 60% ṼO_2_max, 16 s of all-out sprinting, and 14 s of active recovery. The measurements included oxygen uptake (ṼO_2_), heart rate, and power output throughout the trial. The mean and peak power output (PPO), time to peak power, and fatigue index (FI) were assessed during all 16-s sprints. Blood samples were obtained using an antecubital venous indwelling catheter to measure lactate, pH, and bicarbonate (HCO_3_^−^) concentrations at rest and after each sprint. The findings revealed a significant decrease in absolute PPO values during the 8th and 9th sprints in the placebo group, as well as a significant reduction in the relative ΔPPO values during the 6th, 8th, and 9th sprints (mean decrease −12 ± 5%, *p* < 0.006). Conversely, the HRW group exhibited no significant changes in PPO. Mean power, FI, time to peak power, heart rate, and total work did not differ significantly between groups. Lactate levels increased with the number of sprints, while the pH and HCO_3_^−^ gradually decreased in both groups. These results conclusively suggest that two weeks of HRW consumption may effectively preserve PPO during repeated sprints lasting over 30 min [24].

In a different randomized, double-blind study, 22 male amateur middle-distance runners participated, where all subjects ingested either 500 mL of HRW or a placebo (purified water) supplement 30 min prior to the commencement of the trial. Over a span of 4 days, various performance parameters including the maximum aerobic speed and time to exhaustion at maximum aerobic speed during the Vameval test, as well as squat jump, countermovement jump, and quintuple jump test were evaluated. Additionally, the rate of perceived exertion and peak heart rate were monitored during the aerobic testing. The results indicated that HRW consumption led to enhancements in the maximum aerobic speed (*p* = 0.04; ∆ = 0.55%; d = 0.06), an increase in the peak heart rate (*p* < 0.001; ∆ = 1.01%; d = 0.21), and a higher rate of perceived exertion (*p* < 0.001; ∆ = 0.83%; d = 0.14) during the Vameval test compared with the placebo group. Similarly, significant increases were observed in the time to exhaustion at maximum (*p* < 0.001; ∆ = 7.71%; d = 0.39), perceived exertion (*p* < 0.001; ∆ = 6.65%; d = 0.77), and peak heart rate (*p* < 0.001; ∆ = 1.98%; d = 0.31) with HRW intake during the time to exhaustion in the maximal aerobic speed test. However, no statistically significant differences were noted between the HRW and placebo conditions in the squat jump (*p* = 0.120; ∆ = 2.26%; d = 0.10), countermovement jump (*p* = 0.382;∆ = 1.62%; d = 0.07), or quintuple jump test performance (*p* = 0.267; ∆ = 0.57%; d = 0.04). Overall, the findings suggest that the ingestion of 500 mL of HRW resulted in significant improvements in peak heart rate, time to failure, and RPE among amateur endurance athletes, without notable effects on maximal aerobic speed or jump performance [25].

In another investigation, 18 dragon boat athletes, comprising 12 males and 6 females, were enlisted and randomly allocated into two groups: the HRW group (mean age: 23.22 ± 1.09 years; n = 9) and the placebo (purified water) group (mean age: 22.67 ± 0.87 years; n = 9). These athletes, engaged in 4 h of daily training (2 h in the morning and 2 h in the afternoon), were divided into the HRW group and the placebo group, with each group assigned to consume HRW or placebo for a duration of 7 days. Each participant underwent a 30-s rowing ergometer test, with heart rate measurements recorded at baseline (i.e., day 1) and post intervention (day 8). The experimental findings revealed that HRW consumption resulted in an increased maximum power (HRW: pre: 401.00 ± 111.38 w; post: 442.67 ± 112.47 w, *p* < 0.05; placebo water: pre: 390.22 ± 189.97 w, post: 390.11 ± 155.14 w, *p* > 0.05) and average power (HRW: pre: 300.89 ± 91.08 w, post: 321.33 ± 77.47 w, *p* < 0.05; placebo water: pre: 290.78 ± 153.25 w, post: 296.22 ± 123.52 w, *p* > 0.05) output during the 30-s rowing test, accompanied by a reduction in the maximum heart rate (HRW: pre: 176.89 ± 11.36 b/min; post: 162.44 ± 21.39 b/min, *p* < 0.05; placebo water: pre: 162.78 ± 17.22 b/min; post: 164.33 ± 11.31 b/min, *p* > 0.05) during the exercise period. Furthermore, following the rowing test, the HRW group exhibited a significant decrease in heart rate after 2 min of recovery, whereas no such reduction was observed in the placebo water group. Notably, the 30-s rowing distance did not significantly deviate from the predicted 500 m rowing time. In conclusion, the short-term consumption of HRW demonstrated efficacy in enhancing the strength performance of dragon boat athletes, along with facilitating heart rate recovery to baseline levels post exercise. These findings suggest that HRW may represent a suitable hydration strategy for athletes [4].

A randomized, double-blind, placebo-controlled, crossover study involving 37 volunteers was conducted. The participants were divided into two groups: untrained individuals (n = 15; age: 26.3 ± 5.9 years, weight: 69.8 ± 11.4 kg, height: 169.3 ± 7.1 cm, body fat: 24.5 ± 6.5%) and trained amateur cyclists (n = 12; age: 25.5 ± 5.5 years, weight: 70.9 ± 8.5 kg, height: 177.3 ± 6.6 cm, body fat: 17.9 ± 5.8%). Each participant received either placebo (purified water) or HRW (pH 7.5; hydrogen concentration: 1.9 ppm; oxidation/reduction potential: −600 mV). Performance was evaluated using incremental ṼO_2_ max and maximal anaerobic tests at the end of a 7-day intake period. The results indicated that only the trained cyclists showed improved performance in the anaerobic test after HRW ingestion. Specifically, they exhibited increases in peak power (from 766.2 ± 125.6 to 826.5 ± 143.4 W; d = 0.51) and mean power (from 350.0 ± 53.5 to 380.2 ± 71.3 W; d = 0.51), along with a decrease in the fatigue index (from 77.6 ± 5.8 to 75.1 ± 5.9%; d = 0.45). These findings suggest that the ergogenic effect of HRW is influenced by the training state and that a 7-day regimen of HRW ingestion may be an effective strategy to enhance anaerobic performance in trained cyclists [19].

### 3.2. Non-Significant Outcomes

While many studies indicate that HRW has the potential to enhance athletic performance, some findings suggest that its benefits may not be universally significant. For example, two research papers have shown that HRW does not improve the performance of trained track and field athletes running to exhaustion at the maximum aerobic speed, nor does it increase heart rate or race time. These results suggest that HRW may not be effective under certain conditions, highlighting the need for further in-depth research to clarify its role in athletic performance.

In a recent investigation, 24 male runners with a mean age of 17.5 ± 1.8 years, body mass index of 21.0 ± 1.3 kg·m^−2^, and ṼO_2_max of 55.0 ± 4.6 mL·kg^−1^·min^−1^ were recruited for a randomized, double-blind, placebo-controlled crossover study. Participants were instructed to consume 1260 mL of HRW, as 420 mL is the standard size offered by the company, while the placebo group consumed the same quantity of purified water. To avoid stomach discomfort from drinking too much water at once, the intake was divided into four doses: 420 mL HRW 120 min before exercise, 420 mL HRW 60 min before, 210 mL HRW 30 min before, and 210 mL HRW 10 min before. The running protocol consisted of three phases: a 3-min warm-up at 10 km·h^−1^, followed by a 1-min transition phase at an individually determined speed (calculated as the average of 10 km·h^−1^ and maximum aerobic speed), and finally, running at the personal maximum aerobic speed until exhaustion. Time to failure, cardiorespiratory parameters, and post-exercise blood lactate concentration were assessed. The results indicated that HRW administration had no significant impact compared with placebo on various variables when running to failure at the maximum aerobic speed, including time to failure (217 ± 49 vs. 227 ± 53 s, *p* = 0.20), post-exercise blood lactate concentration (9.9 ± 2.2 vs. 10.1 ± 2.0 mmol·L^−1^, *p* = 0.42), maximum heart rate (186 ± 9 vs. 186 ± 9 beats·min^−1^, *p* = 0.80), and oxygen uptake (53.1 ± 4.5 vs. 52.2 ± 4.7 mL·kg^−1^·min^−1^, *p* = 0.33). Additionally, none of the variables examined as potential moderators were significantly correlated with time to failure (Spearman correlation coefficients ranged from −0.28 to 0.30, all *p* ≥ 0.16). In conclusion, the pre-exercise administration of 1260 mL of HRW did not exhibit a potentiating effect on the performance of trained track and field athletes running to failure at the maximum aerobic speed [26].

A study involving 16 male participants (mean ± SD: age 31.6 ± 8.6 years; ṼO_2_max 57.2 ± 8.9 mL·kg^−1^·min^−1^; body fat 13.4% ± 4.4%) employed a randomized, double-blind, placebo-controlled crossover design experiment. The participants were administered either HRW or placebo (purified water) prior to engaging in two 4.2-km uphill races conducted one week apart. The race time, average race heart rate, and perceived exertion immediately after each race were evaluated. The analysis of data from all runners did not yield clear findings regarding the effect of HRW on race time (−10 to 7 s, 90% confidence interval) or heart rate (−2 to 3 beats·min^−1^). Moreover, the impact on the post-race perceived exertion rating ranged from −0.1 to 1.0. Additionally, a negative correlation between the race time difference and mean race time (r = −0.79 to −0.15) was observed. Specifically, HRW intake seemed to decrease the race time for the four slowest runners (race time = 1490 ± 91 s) by −36 to −3 s, while its effect on the four fastest runners (race time = 1069 ± 53 s) ranged from −10 to 26 s, showing uncertainty. Overall, the influence of HRW intake on fatigue during athletic performance remains inconclusive when considering the mean group values. However, it appears that the effectiveness of HRW in reducing fatigue during exercise may vary based on the individual running ability [27].

While many studies have shown that HRW has the potential to improve athletic performance, some findings suggest that it may not lead to significant improvements. Therefore, further comprehensive investigations are necessary to clarify this issue. In summary, current studies have reached varying conclusions on the efficacy of HRW in enhancing athletic performance, highlighting the need for future exploration and refinement of experimental methods.

## 4. Potential Mechanisms of Action

HRW, also referred to as hydrogenated water, is ordinary water infused with molecular hydrogen [1]. Molecular hydrogen is introduced into the water by dissolving it under high pressure, leading to the formation of a supersaturated solution [28]. Due to the small sizes of hydrogen molecules, they can easily permeate water and remain dissolved for an extended duration. Hence, hydrogen serves as the primary agent responsible for the effects observed in HRW [29].

HRW can be produced either by dissolving H_2_ gas in water under high pressure or by using hydrogen-generating tablets. Under the standard ambient temperature and pressure, the solubility of H_2_ in water is 1.56 mg/L. Although 1.6 mg of H_2_ per liter may seem minimal, the “therapeutic moles” of H_2_ at this concentration are greater than those of vitamin C in a 100 mg dose (0.78 mmol vs. 0.56 mmol) due to the difference in molar mass. Additionally, some hydrogen-producing tablets can supersaturate the water, providing over 5 mg of H_2_ per tablet. In some cases, consuming HRW may yield more significant effects than inhaling hydrogen gas, even when the hydrogen dose in the water is lower. Peak hydrogen levels are typically reached within 5–15 min after ingestion and return to baseline within 45–90 min, depending on the dose administered.

Although numerous studies and literature reports have demonstrated the effectiveness of HRW in enhancing athletic performance, expediting recovery, and alleviating exercise-induced fatigue, the mechanisms underlying these benefits are broad and lack precise, one-to-one explanations. Furthermore, these effects often occur concurrently and are interconnected. The principal mechanisms through which HRW confers these benefits can be outlined as follows.

### 4.1. HRW as a Selective Antioxidant

One of the most significant properties of hydrogen molecules is their ability to act as selective antioxidants [30]. One study has shown that oxidative stress originates from an excess of reactive oxygen species or cells with a strong oxidative potential containing free radicals [31]. Most superoxide anion radicals are generated by leakage in the electron transport chain or the Krebs cycle, and metabolic oxidases also produce superoxide anion radicals [32]. Superoxide dismutase converts these radicals into hydrogen peroxide, which is then broken down into H_2_O [32]. However, excess superoxide anion radicals can reduce the Fe^3+^ and Cu^2+^ ions. The reduced metal ions produce hydroxyl radicals through the Fenton reaction with hydrogen peroxide [33]. Hydroxyl radicals are strongly oxidizing. To study their detoxification system, researchers treated cells to rapidly convert superoxide anion radicals into hydrogen peroxide, and dissolved H_2_ and O_2_ in the cells. Subsequently, H_2_ and O_2_ were dissolved in the culture medium. The results showed that H_2_ specifically reduced the levels of hydroxyl radicals. Furthermore, the researchers pretreated the cells with Cu^2+^ and added ascorbic acid to promote the production of hydroxyl radicals from hydrogen peroxide, reducing the conversion of Cu^2+^ to Cu^+^ [33]. This induced the intracellular production of hydroxyl radicals through the Fenton reaction, directly confirming the ability of H_2_ to protect cells from hydroxyl radicals [34]. Unlike other antioxidants that may indiscriminately neutralize both harmful and beneficial reactive species, hydrogen molecules specifically target the most harmful reactive oxygen species and reactive nitrogen species, such as hydroxyl radicals and peroxynitrite [30]. ROS and RNS are highly reactive molecules generated within cells and play crucial regulatory roles [35]. However, the excessive production or imbalance of ROS and RNS can have adverse effects on cellular function, potentially leading to severe cellular damage [36].

The consequences of excessive ROS and RNS production include oxidative stress and cellular damage [37]. Oxidative stress arises from an imbalance in the redox process, elevating oxidant levels within cells and causing damage to proteins, nucleic acids, and lipids, ultimately leading to cell dysfunction or apoptosis [31]. Additionally, ROS- and RNS-induced DNA damage can result in mutations, increasing the risk of multiple diseases [38].

Protein oxidation due to ROS and RNS can lead to structural and functional abnormalities, disrupting the normal cellular metabolism and signaling pathways, and potentially contributing to disease development [39]. Moreover, excessive ROS and RNS can interfere with intracellular signaling pathways, impacting cellular processes such as proliferation, apoptosis, and inflammation regulation [40]. Furthermore, ROS and RNS involvement in inflammation and immune responses, when excessive, can exacerbate inflammatory conditions or trigger immune-related diseases [41].

Meanwhile, the preservation of ROS involved in physiological signaling offers several benefits. Cellular homeostasis: Certain ROS, at low levels, are essential for maintaining cellular homeostasis [42]. They participate in redox signaling, which regulates various cellular processes, including metabolism, cell growth, and apoptosis [43]. By preserving these signaling molecules, hydrogen helps to maintain the balance between oxidants and antioxidants, which is crucial for normal cellular function [44].

Adaptation to physical stress: During exercise, the body naturally produces ROS, which act as signaling molecules to initiate adaptive responses [45]. These adaptations include enhanced mitochondrial biogenesis, improved antioxidant defenses, and increased muscle endurance [46]. By selectively targeting harmful ROS while sparing those involved in signaling, hydrogen supports these beneficial adaptations without disrupting the essential physiological processes [47].

Improved cellular communication: ROS are involved in intercellular communication, particularly in immune responses and the regulation of inflammation [48]. By preserving these ROS, hydrogen can support proper immune function and inflammatory responses, helping the body to respond effectively to injuries or infections while minimizing unnecessary inflammation that can lead to tissue damage [49].

Enhanced recovery: The selective scavenging of harmful ROS can reduce the muscle damage and inflammation caused by intense exercise, while the preservation of signaling ROS can enhance the recovery processes. This balance helps athletes to recover faster and more effectively, improving overall training outcomes [50].

Therefore, managing ROS and RNS production and removal is crucial for maintaining cellular and tissue health and reducing the risk of various diseases, including cancer, cardiovascular disease, and inflammatory disorders [51]. The rapid diffusion of hydrogen molecules across cell membranes enables them to effectively reach the intracellular compartments where ROS and RNS are generated, which is particularly important during heightened reactive species production, such as during exercise [52]. By selectively scavenging harmful reactive species, hydrogen molecules help to maintain redox homeostasis, preserving cellular integrity and supporting optimal physiological function during and after physical exertion [53].

### 4.2. Effects of HRW on Exercise through Oxidative Stress Mechanisms

Oxidative stress typically occurs in the body when external stimuli induce the production of reactive substances that exceed the body’s antioxidant capacity. During exercise, particularly high-intensity exercise, the increased metabolic rate leads to the heightened production of ROS and RNS. These reactive substances can cause oxidative modifications to lipids, proteins, and DNA, resulting in cellular dysfunction and damage [54].

#### 4.2.1. Muscle Fatigue

One of the primary effects of oxidative stress is muscle fatigue. During muscle contraction, mitochondria are the main source of ROS generation [55]. Additionally, enzyme systems such as NADPH oxidase, xanthine oxidase, and cytochrome P450 also contribute to ROS production [56]. When the exercise intensity or duration increase, the rate of ROS generation can exceed the clearance capacity of the intracellular antioxidant system, leading to ROS accumulation.

ROS can impair muscle fiber function by disrupting calcium homeostasis and damaging mitochondrial proteins, which results in reduced ATP production. For example, ROS can oxidize calcium ion channels and pumps on cell membranes, such as L-type calcium channels, calcium-release channels (e.g., Ryanodine receptors of the sarcoplasmic reticulum), and calcium pumps (e.g., Sarco/Endoplasmic Reticulum Calcium ATPase (SERCA)) [57]. This oxidative damage can lead to: 1. Abnormal function of calcium channels, resulting in increased calcium influx [58]; 2. Reduced activity of the SERCA pump, preventing the effective recycling of calcium ions from the cytoplasm to the sarcoplasmic reticulum [59]; 3. Modifications to calcium-binding proteins such as calmodulin and troponin, affecting their ability to bind calcium ions and thereby interfering with the contraction and relaxation cycle of muscle fibers [60].

Similarly, mitochondria, the cell’s energy factories, are responsible for generating ATP. ROS can cause oxidative damage to mitochondrial DNA (mtDNA), triggering mutations and changes in gene expression. Since mtDNA encodes various key mitochondrial proteins, including those involved in the electron transport chain, this damage can directly affect ATP synthesis. ROS can also oxidize the respiratory chain complexes (such as complexes I, III, and IV) on the inner mitochondrial membrane, leading to: 1. Decreased activity of these enzymes and blocked electron transfer, reducing ATP generation [61]; 2. Reduced efficiency of oxidative phosphorylation [62]; 3. Increased electron leakage, which further generates ROS, creating a vicious cycle [63].

ROS-induced peroxidation of lipids in the inner mitochondrial membrane can destroy membrane integrity and function, leading to: 1. Loss of membrane potential, affecting the driving force for ATP synthesis [64]; 2. Release of cytochrome c from mitochondria, triggering apoptosis signaling pathways [65].

Moreover, oxidative damage to ATP synthase by ROS directly affects ATP generation efficiency. ATP synthase, a key enzyme on the inner mitochondrial membrane, when damaged, leads to: 1. Reduced ATP production and an insufficient energy supply to muscle fibers [66]; 2. Energy metabolism disorders, affecting muscle contraction and recovery, eventually resulting in decreased muscle contraction efficiency and endurance [67].

#### 4.2.2. Inflammation

Oxidative stress is also closely linked to inflammation. Reactive oxygen species (ROS) can activate various inflammatory signaling pathways, leading to the production of pro-inflammatory cytokines [68]. These cytokines, such as tumor necrosis factor-alpha (TNF-α), interleukins (e.g., IL-1β, IL-6), and interferons, are crucial mediators of the inflammatory response [69]. When produced in excess, they can contribute to the development and persistence of chronic inflammation.

Chronic inflammation, in turn, can exacerbate muscle damage and delay recovery, impacting overall performance [70]. This prolonged inflammatory state can lead to a cycle where muscle repair is hindered, resulting in further damage and additional inflammation. Over time, this can lead to a decline in muscle function and strength, impairing an athlete’s ability to train and compete effectively [71].

Moreover, chronic inflammation has been implicated in various metabolic and degenerative diseases, such as insulin resistance, cardiovascular diseases, and neurodegenerative conditions [72]. In the context of sports and physical performance, managing oxidative stress and inflammation is therefore crucial not only for immediate recovery but also for long-term health and performance sustainability.

HRW, working as an important antioxidant, plays a critical role in mitigating oxidative stress and its inflammatory consequences by targeting ROS and supporting the body’s defense system.

#### 4.2.3. Performance Decline

The cumulative effects of oxidative stress, including muscle fatigue and inflammation, contribute to a decline in athletic performance. This is particularly critical during prolonged or repeated bouts of high-intensity exercise, where maintaining performance is essential [73].

During high-intensity exercise, the body experiences a significant increase in metabolic activity, leading to the enhanced production of reactive oxygen species (ROS). While ROS play a role in cellular signaling and homeostasis, their excessive accumulation can overwhelm the body’s antioxidant defenses, resulting in oxidative stress. This stress damages cellular components, such as lipids, proteins, and DNA, impairing muscle function and overall performance [74].

Muscle fatigue is a direct consequence of oxidative stress. ROS can interfere with the excitation–contraction coupling process in muscle fibers, disrupt calcium homeostasis, and impair mitochondrial function, all of which are crucial for muscle contraction and endurance [46]. As a result, athletes may experience reduced strength, power output, and endurance, limiting their ability to perform at optimal levels during extended periods of physical exertion [75].

Inflammation further exacerbates performance decline. Chronic inflammation induced by oxidative stress can lead to persistent muscle soreness, stiffness, and injury [76]. This not only affects an athlete’s immediate performance but also hinders recovery, making it difficult to maintain consistent training schedules. Over time, the accumulation of unresolved inflammation can lead to chronic conditions such as tendinitis, arthritis, and other musculoskeletal disorders, further diminishing athletic performance [77].

The negative impact of oxidative stress and inflammation on performance is not limited to physical capabilities [78]. Mental fatigue and decreased cognitive function are also associated with high levels of oxidative stress [79]. Athletes may experience difficulties in concentration, decision-making, and reaction time, all of which are critical for success in competitive sports.

HRW, acting as an important antioxidant, can help to bolster the body’s defenses against ROS and reduce oxidative damage, which can further alleviate the decline in performance caused by ROS.

### 4.3. Roles of HRW in Redox Homeostasis

By selectively targeting the most harmful reactive species, molecular hydrogen plays a crucial role in restoring redox homeostasis. This balance is essential for maintaining cellular function and overall health. The protective effects of hydrogen can be attributed to several key mechanisms.

#### 4.3.1. Scavenging of Hydroxyl Radicals and Peroxynitrite

Hydrogen efficiently neutralizes the highly reactive hydroxyl radicals and peroxynitrite, which are not adequately targeted by other endogenous antioxidants [80]. Hydroxyl radicals are extremely reactive and can damage virtually all types of biomolecules at the site of formation, making them one of the most dangerous forms of reactive oxygen species [81]. Peroxynitrite, a potent oxidant and nitrating agent formed from the reaction of nitric oxide with superoxide, can also cause significant damage to a wide array of cellular components [82].

The ability of the hydrogen in HRW to specifically target and neutralize these reactive species is crucial, as it reduces the oxidative burden on cells. This protective action helps to prevent oxidative damage to critical cellular components such as DNA, proteins, and lipids [83]. DNA damage can lead to mutations, genomic instability, and potentially carcinogenesis. Protein oxidation can result in the loss of enzyme activity, structural alterations, and impaired cellular functions [84]. Lipid peroxidation can compromise the integrity of cell membranes, affecting membrane fluidity and permeability, and ultimately leading to cell death [85].

By scavenging hydroxyl radicals and peroxynitrite, hydrogen helps to maintain the structural and functional integrity of cellular components [86]. This action not only protects cells from immediate oxidative damage but also supports long-term cellular health by preventing the accumulation of oxidative damage over time [87]. The reduction in oxidative stress can also mitigate the onset of various oxidative stress-related diseases, including neurodegenerative disorders, cardiovascular diseases, and chronic inflammatory conditions [88].

Furthermore, hydrogen’s selective scavenging mechanism does not interfere with the signaling roles of other reactive oxygen and nitrogen species, which are vital for normal cellular processes. This selective neutrality ensures that hydrogen does not disrupt the essential cellular functions while providing its protective effects. Thus, the unique properties of HRW make it an effective and safe antioxidant for maintaining cellular homeostasis and promoting overall health.

#### 4.3.2. Regulation of Antioxidant Enzymes

Hydrogen can modulate the expression and activity of endogenous antioxidant enzymes, such as superoxide dismutase (SOD), catalase, and glutathione peroxidase. These enzymes are pivotal in detoxifying reactive species and maintaining the redox balance [89].

SOD is essential for the dismutation of superoxide radicals into oxygen and hydrogen peroxide, reducing the harmful effects of superoxide radicals on cellular components [90]. Catalase then converts hydrogen peroxide, which can be harmful in high concentrations, into water and oxygen, thus preventing the potential damage from hydrogen peroxide accumulation [91]. Glutathione peroxidase further assists in reducing hydrogen peroxide and other peroxides, using glutathione as a substrate, thereby protecting the cell from oxidative damage [92].

By upregulating these enzymes, hydrogen enhances the cellular defense mechanisms against oxidative stress [93]. Increased levels of SOD, catalase, and glutathione peroxidase lead to a more efficient detoxification process, allowing cells to better manage and neutralize ROS and other free radicals [81]. This upregulation ensures a more robust and sustained antioxidative response, which is crucial for the longevity and health of the cells.

Moreover, the modulation of these enzymes by hydrogen can contribute to the repair and recovery of damaged tissues [94]. Enhanced antioxidative enzyme activity supports cellular resilience, allowing cells to withstand and recover from oxidative insults more effectively. This can be particularly beneficial in conditions that are characterized by chronic oxidative stress, demonstrating beneficial effects on the nervous system, cardiovascular system, and anti-inflammatory processes [95].

Furthermore, the regulation of antioxidant enzymes by hydrogen does not interfere with the necessary signaling functions of ROS in normal cellular processes, such as cell proliferation, apoptosis, and immune responses [52]. This selective regulation ensures that while the harmful effects of excessive ROS are mitigated, their beneficial roles are preserved.

Overall, hydrogen’s ability to modulate endogenous antioxidant enzymes underscores its potential as a therapeutic agent in oxidative stress-related conditions. By enhancing the body’s natural antioxidative defenses, hydrogen helps to maintain redox homeostasis, supports cellular health, and protects against a wide range of diseases linked to oxidative damage.

#### 4.3.3. Mitigation of Lipid Peroxidation

Lipid peroxidation, the oxidative degradation of lipids, is a major consequence of oxidative stress that affects cell membranes and other lipid-containing structures [96]. The antioxidative action of hydrogen helps to prevent lipid peroxidation, thereby preserving the integrity and functionality of cellular membranes [97].

This preservation is critical because it maintains membrane fluidity and permeability, which are essential for proper cell signaling and nutrient transport. Additionally, intact cellular membranes serve as barriers against harmful substances and pathogens, safeguarding the internal environment of cells [98].

Furthermore, lipid peroxidation can lead to the formation of reactive aldehydes, such as malondialdehyde (MDA) and 4-hydroxynonenal (4-HNE), which are highly reactive and can modify cellular proteins and nucleic acids [99]. By inhibiting lipid peroxidation, hydrogen reduces the generation of these reactive aldehydes, thus minimizing their damaging effects on cellular components [100].

In conclusion, the ability of hydrogen to mitigate lipid peroxidation underscores its importance in maintaining cellular function and health. By preserving membrane integrity and reducing the formation of reactive aldehydes, hydrogen contributes to cellular homeostasis and may offer protection against oxidative stress-related diseases. Further research into the mechanisms underlying hydrogen’s antioxidative effects on lipid peroxidation will enhance our understanding of its therapeutic potential.

#### 4.3.4. Reduction of Inflammatory Responses

Oxidative stress often triggers inflammatory responses, which can exacerbate cellular damage. Hydrogen’s capacity to modulate oxidative stress also results in decreased inflammation [101].

Hydrogen effectively mitigates the inflammatory cascade by scavenging ROS and restoring the redox balance. This leads to the reduced activation of transcription factors, such as nuclear factor kappa B (NF-κB), and the diminished expression of pro-inflammatory cytokines, including TNF-α, IL-6, and IL-1β [102].

Moreover, hydrogen has been demonstrated to upregulate anti-inflammatory mediators such as interleukin-10 (IL-10) and heme oxygenase-1 (HO-1), which contribute to inflammation resolution and tissue repair processes [103]. By reducing levels of pro-inflammatory cytokines and inhibiting inflammatory pathways, hydrogen helps to alleviate chronic inflammation [104].

Overall, hydrogen’s anti-inflammatory properties, combined with its antioxidative effects, position it as a promising therapeutic agent for addressing oxidative stress-related inflammatory diseases. Further research into the mechanisms underlying hydrogen’s anti-inflammatory actions will provide valuable insights into its potential clinical applications and therapeutic efficacy.

#### 4.3.5. Protection against Mitochondrial Dysfunction

Mitochondria, often referred to as the powerhouse of the cell, are particularly vulnerable to oxidative stress due to their high metabolic activity and the presence of electron transport chains [105]. Oxidative stress can impair mitochondrial function by damaging proteins, lipids, and DNA within these organelles [106]. This damage can lead to reduced ATP production and the increased generation of ROS, further exacerbating oxidative stress and cellular damage [107].

Hydrogen’s antioxidative properties play a crucial role in protecting mitochondria from oxidative damage [108]. By scavenging ROS and restoring the redox balance, hydrogen helps to preserve the integrity of mitochondrial components and maintain their functionality [109]. This includes preserving the integrity of mitochondrial membranes, sustaining the activity of electron transport chain complexes, and safeguarding mitochondrial DNA from oxidative lesions [110].

Moreover, hydrogen has been shown to upregulate antioxidant enzymes within mitochondria, such as manganese superoxide dismutase and glutathione peroxidase, which further contribute to mitochondrial protection [111]. By enhancing the cellular antioxidant defense system, hydrogen ensures efficient ROS detoxification within mitochondria, thereby reducing the risk of mitochondrial dysfunction [112].

This mitochondrial protection is crucial for sustaining cellular energy metabolism and overall cell viability [113]. Mitochondria play a central role in ATP synthesis through oxidative phosphorylation, providing the energy necessary for cellular processes [114]. By preserving mitochondrial function, hydrogen helps to maintain adequate ATP levels, supporting essential cellular functions such as cell growth, proliferation, and the maintenance of membrane potential [108,114,115].

In summary, hydrogen’s ability to protect against mitochondrial dysfunction underscores its importance in maintaining cellular energy metabolism and overall cell viability.

#### 4.3.6. Regulation of Cellular Signaling Pathways

Beyond its antioxidative effects, hydrogen also influences various cellular signaling pathways that are involved in cell survival, growth, and repair. By modulating these pathways, hydrogen promotes cellular resilience and adaptation to stress, further supporting redox homeostasis and overall cellular health [7].

One key pathway affected by hydrogen is the nuclear factor erythroid 2–related factor 2 (Nrf2) pathway, which plays a pivotal role in the cellular antioxidant response. The activation of Nrf2 leads to the upregulation of various cytoprotective genes, including those encoding for antioxidant proteins and phase II detoxifying enzymes. Western blotting of Nrf2 in melanocytes and keratinocytes was performed in this study by incubating the cells with or without 75% H_2_ for 24 h, followed by 12 h of incubation in an environment with or without 1 mM hydrogen peroxide. Hydrogen has been shown to enhance Nrf2 activity, thereby boosting the cell’s ability to combat oxidative stress and detoxify harmful substances [116].

Additionally, hydrogen influences the PI3K/Akt pathway, a critical regulator of cell growth and survival. The activation of this pathway by hydrogen can promote cell proliferation and protect against apoptosis in response to oxidative stress. This pro-survival signaling helps to maintain tissue integrity and function under stressful conditions [117].

Hydrogen also modulates the mitogen-activated protein kinase (MAPK) signaling pathways, which are involved in regulating cell differentiation, proliferation, and responses to external stimuli. By fine-tuning these pathways, hydrogen aids in the cellular adaptation to environmental changes and stressors, promoting overall cellular health and longevity [118].

Moreover, hydrogen has been found to impact the AMP-activated protein kinase (AMPK) pathway, a crucial energy sensor in cells. The activation of AMPK by hydrogen enhances cellular energy metabolism, promoting efficient ATP production and utilization. This ensures that cells have the energy needed for repair and maintenance processes, especially under conditions of stress [119].

In summary, molecular hydrogen’s multifaceted role in redox homeostasis encompasses the scavenging of harmful reactive species, regulation of antioxidant enzymes, prevention of lipid peroxidation, reduction of inflammatory responses, protection of mitochondrial function, and enhancement of cellular signaling pathways [7]. These combined actions underscore the potential of hydrogen as a therapeutic agent in mitigating oxidative stress-related diseases and promoting overall health. Through its diverse mechanisms of action, hydrogen not only protects cells from damage but also supports their repair and adaptive capabilities, highlighting its significance in the maintenance of cellular and systemic health.

## 5. Future Perspectives

The potential of HRW to enhance athletic performance and recovery offers a promising avenue for future research and application. Current studies highlight its selective antioxidant properties and benefits on various aspects of athletic performance; however, several areas warrant further exploration.

First, quantifying the amount of hydrogen ingested during the consumption of HRW is crucial in understanding its role as an effective active substance.

Second, the precise mechanisms by which hydrogen functions need further investigation. Understanding the biochemical pathways involved in the clearance of reactive oxygen species and reactive nitrogen species, as well as the regulation of inflammatory responses and mitochondrial function, will provide clearer insights into the benefits of HRW for athletes. These mechanistic studies should not only examine its disease-treating mechanisms but also focus more on its potential to enhance athletic performance.

Additionally, to effectively market HRW and encourage its use among a broad range of sports participants, future research should address the long-term effects of HRW supplementation. It is essential to determine whether the benefits of HRW are sustained with prolonged use or if they diminish over time. Investigating the potential side effects or contraindications is also critical to ensuring that HRW is safe for all athletes. Only safe and reliable HRW can be widely recommended to athletes and the sports community.

Innovative applications of HRW in sports nutrition and recovery products can further improve accessibility and convenience for athletes. Exploring its synergy with other supplements and training interventions may yield new strategies for optimizing athletic performance.

Finally, interdisciplinary collaborations between sports scientists, biochemists, and medical professionals will be key to advancing our understanding of HRW. Such collaborations could facilitate the development of new technologies to monitor oxidative stress and recovery, ultimately providing athletes with more personalized and effective training plans, allowing HRW to complement professional training regimens and achieve the best results.

In summary, while HRW holds great promise for enhancing athletic performance and recovery, further research is essential to fully realize its potential. Addressing the current knowledge gaps and exploring new applications will pave the way for HRW to become an integral part of sports science and nutrition.

Meanwhile, this article has some limitations. For instance, the current body of literature on HRW and its effects on athletic performance remains limited, with only nine relevant studies available, which may not provide comprehensive insights. We hope that future research will expand the number of studies in this field, offering a broader reference base. Additionally, this article does not establish the specific molecular mechanisms through which HRW enhances athletic performance. We anticipate that future key studies will identify core target molecules and clarify how HRW modulates these molecules to effectively improve athletic performance.

## 6. Conclusions

This comprehensive review of HRW underscores its significant potential in enhancing athletic performance and recovery. Molecular hydrogen, the active component in HRW, exhibits antioxidant properties that play a crucial role in mitigating oxidative stress, reducing inflammation, and preserving mitochondrial function. These combined effects contribute to improved endurance, accelerated recovery, reduced muscle fatigue, and enhanced overall performance.

Molecular hydrogen selectively targets harmful reactive oxygen and nitrogen species without disrupting essential physiological signaling, distinguishing it from traditional antioxidants. Its rapid diffusion across cell membranes further supports mitochondrial health by preventing oxidative damage and enhancing ATP production. Additionally, HRW’s anti-inflammatory benefits are demonstrated through the modulation of pro-inflammatory cytokines and signaling pathways, which can support overall athletic performance.

Despite the promising initial findings, extensive clinical trials are necessary. Future research should focus on standardizing dosing protocols, understanding the long-term effects, and investigating the potential side effects. Further exploration of the specific biochemical pathways involved will provide deeper insights into HRW’s mechanisms of action.

In conclusion, HRW presents a promising avenue for sports science and nutrition. With further research and validation, HRW could become a valuable tool for athletes seeking to optimize performance and recovery.

## Figures and Tables

**Table 1:** Summary of studies on the effects of HRW in exercise.

No. [Ref.]	Number of Subjects	Dosage (mL)	Findings
1 [17]	16	1260	The study observed an improvement in sprint times, while the lactate concentration and perceived exertion ratings remained unchanged.
2 [22]	12	1260	The study observed improvements in lunges and muscle function, a reduction in the lactate response, and the alleviation of delayed onset muscle soreness.
3 [2]	12	2520	The study observed improvements in countermovement jump height and reductions in creatine kinase blood activity and muscle soreness following training.
4 [23]	8	2000	The study observed enhancements in mean and peak power output, time to peak power, fatigue index, and total work capacity.
5 [24]	22	500	The study observed improvements in maximal aerobic speed during the Vameval test, time to exhaustion at the maximal aerobic speed, perceived exertion rate, and peak heart rate. However, no significant changes were found in squat jump, countermovement jump, and five jump test performance.
6 [4]	18	1000	The study observed improvements in both the maximum and average power during a 30-s rowing test, along with a decrease in maximum heart rate during the test period. Additionally, heart rate dropped significantly after 2 min of recovery.
7 [18]	37	1920–2240	The study observed performance improvements in trained cyclists, including increased peak and mean power outputs, along with a decreased fatigue index in the anaerobic test.
8 [25]	24	1260	The study observed improvements in time to exhaustion, post-exercise blood lactate concentration, maximal heart rate, and oxygen uptake. However, no assessed variables were significantly correlated with time to exhaustion.
9 [26]	16	1680	The study observed no significant changes in race time, average race heart rate, and the rating of perceived exertion immediately after the race.

## Data Availability

No new data were created or analyzed in this study.
